# The “Casting Couch” Scenario: Impact of Perceived Employment Benefit, Reporting Delay, Complainant Gender, and Participant Gender on Juror Decision-Making in Rape Cases

**DOI:** 10.1177/0886260520966679

**Published:** 2020-10-21

**Authors:** Shona Mcintosh, Josh P. Davis

**Affiliations:** University of Greenwich, London, UK

**Keywords:** casting couch, jury decision-making, juror decision making, rape myths, sexual assault

## Abstract

Recent legal and media reports of contemporary and historical rape and sexual assault cases have focused on the entertainment industry, particularly around the notion of the “casting couch.” This scenario, in which a powerful figure obtains sometimes nonconsensual sexual acts from subordinate actors in exchange for employment, was used to explore the influence of rape myths and Sexual Economics Theory on mock-juror decision-making. Participant-jurors (*n* = 907) viewed video and written testimony of a complainant, accusing a male producer of rape. Complainant gender (male, female), delay before reporting the incident to the police (immediately, 6 months, 10 years), and complainant casting in the production were randomly varied (acting role secured, not secured). The strongest effects were that females (79.7%) were significantly more likely than males (62.7%) to deliver a guilty verdict and to recommend longer prison sentences for the offence. When the complainant did not secure the acting role, and they delayed reporting the incident for six months, there was an interaction between complainant gender and verdict. No interacting complainant gender effects on trial outcomes were found in the other delay conditions, or when the actor secured employment. Defendant guilt attributions to the male and female complainant were also differently influenced by rape myth belief levels and homophobic attitudes, but not beliefs in a just world. The casting couch euphemism, reported worldwide, suggests industry acceptance, and may sanitize the act of demanding sex and even committing rape. However, these results have important implications for any occupational setting in which men in positions of power may sexually exploit junior staff.

The “casting couch” cliché of a powerful man obtaining sexual acts from subordinate actors in exchange for employment has been in use for almost a century (Zimmer, 2017). It has featured in media reports of multiple contemporary and historical rape and sexual assault allegations against entertainment industry figures (e.g., Davies & Khomani, 2018; Neumeister, 2018; North, 2018). Allegations have been linked to the #*MeToo* phenomenon. Millions of women claimed to have been victims of sexual harassment and rape, driven by employer–employee workplace power imbalances (Farrow, 2017; *Washington Post*, 2017). This form of “quid pro quo” sexual harassment encompasses many behaviors including rape (e.g., Siuta & Bergman, 2019), and even if rape is not committed, it is an offence in many countries including the UK (Singh, 1998) and the European Union (European Commission and Parliament, 2006). Due to low reporting and conviction levels, no accurate information on prevalence of sexual harassment in its widest sense nor the specific quid pro quo type exists in the UK (House of Commons Women and Equalities Committee, 2018) or Europe (Directorate General for Internal Policies of the Union, 2018).

The entertainments industry may be unique in the use of the casting couch trope. The term’s ubiquity suggests tacit approval and may even sanitize acts of demanding nonconsensual sex in exchange for acting roles (Fallon, 2017). Perceived sanction may link to rape myths; popularly held false beliefs which blame, or hold responsible, victims for rape (e.g., Burt, 1980). Rape myths bias police investigations (e.g., Shaw et al., 2017) and jury decision-making (e.g., Dinos et al., 2015; Hockett et al., 2016; Osborn et al., 2018; Waterhouse et al., 2016). It is important that empirical research investigates potential biases of this type, and this research examined whether commonly reported features of the casting couch scenario might impact UK juror decision-making in alleged rape cases.

Rape myths are “prejudicial, stereotyped, or false beliefs about rape, rape victims, and rapists” (Burt, 1980, p. 217). Rape myth acceptance is associated with beliefs that “genuine rape” has several hallmarks. “Real” victims are female, sober, modestly dressed, attacked by a stranger at night in a public place, display resistance-linked injuries, and contact police immediately (Hockett et al., 2016; Waterhouse et al., 2016). However, most rapes do not match these beliefs, and rape myth acceptance shifts blame from the rapist to the victim. Four broad categories have been proposed (Dinos et al., 2015). First, they blame the victim, so that in the casting couch scenario this might link with the victim visiting a “notorious” sexual harasser’s private space (see Gray, 2015). Second, they justify the rapist’s actions (i.e., by the alleged perpetrator believing the victim’s visit was sending an inviting message). Third, they doubt allegation veracity (i.e., by the victim not immediately contacting police; see also Ellison & Munro, 2009; Smith & Skinner, 2017). Fourth, they suggest that rape only happens to certain victims (i.e., those engaging in high-risk, low-morality lifestyles). This final myth is commonly linked to substance abuse and sex work (Grubb & Turner, 2012), and within the casting couch scenario might be interpreted as being an “occupational hazard,” particularly if the actor is offered the desired role.

The high-status aggressor verses lower-status job-seeker power dynamic may trigger rape myths differently dependent on complainant and juror gender. *Sexual Economics Theory* (Baumeister & Vohs, 2004) proposes that unlike males, female sexuality has inherent economic value. Basow and Minieri (2011; see also Rudman & Fetterolf, 2014) argue that rape of females can be perceived as a form of theft. For example, in a date rape context, when a male pays for an expensive night, males—who tend to believe rape myths more than females—place significantly less blame on the rapist than dates where the cost is split or the date is cheap (e.g., Davies et al., 2012; McGee et al., 2011). Females show no such distinction, and these gender effects are also independent of rape myth acceptance levels.

Borcherding and Filson (2001) suggest that unless an actor possesses a high profile, the ability of most to play any part is uncertain. Actor supply likely exceeds demand. Under Sexual Economics Theory, producers may implicitly expect to base casting decisions on whether sexual favors are offered, and if not offered, to coerce sexual activity physically or emotionally. Applying these assumptions to rape myths, if the female complainant secures the acting role, and therefore benefits from the sexual encounter, it might be expected that jurors, particularly males, will assign less blame to the defendant. Conversely, if the female complainant is raped, but not employed, more blame may be directed at the defendant.

Rape myth acceptance correlates with greater victim blame towards males than females (Davies et al., 2012; Russell & Hand, 2017), attitudes linked to homophobia, and beliefs in traditional gender roles (Davies et al., 2012; Lowe & Rogers, 2017; van der Bruggen & Grubb, 2014). Male victims are assumed to be better able to physically resist (van der Bruggen & Grubb, 2014) and are less psychologically impacted by sexual assault (McGee et al., 2011). As Sexual Economics Theory suggests male sexuality is of lower value, whether the male secures the acting role or not following a casting couch rape might be less likely to impact juror attributions.

Where there is no corroborating evidence, rape and sexual assault trial outcomes depend on jury members believing either the complainant’s or defendant’s version of events (Willmott et al., 2018). The jury must decide whether the defendant possessed a reasonable belief that consent was given (Ellison & Munro, 2009). Defense lawyers often draw on rape myths to discredit victims (Smith & Skinner, 2017). It is likely that they would encourage jurors to draw on casting couch ubiquity as indicating complainant compliance and defendant assumptions of implicit consent.

The current study therefore employed a mock-juror decision-making paradigm, based on evidence gathering procedures in England and Wales, to examine whether guilt attributions (verdicts and recommended prison sentences) would be influenced by common casting couch scenario factors. Outcomes of mock-juror research match those of real trials (Bornstein, 1999), and the paradigm allows variables of interest to be examined in order to measure attitudes and biases of members of the public – who may be randomly selected to serve on juries. Those attitudes are likely shared with many individuals working within the criminal justice system regardless of legal jurisdiction and thus outcomes provide insight into whether interventions are required to reduce any biases.

Therefore, mock-jurors, eligible for UK jury service (Gov.UK, 2017) viewed videoed testimony of the male or female complainant describing meeting a male producer in their office and being subsequently raped. The complainant reported whether they gained the acting role or not, and how long they waited to report the offence to the police. Delays, which also trigger rape myths (e.g., Ellison & Munro, 2009; Smith & Skinner, 2017), are a common feature of these cases; and the reported delay was either 1 day, 6 months, or 10 years. Participants also completed the *Acceptance of Modern Myths About Sexual Aggression Scale* (AMMSA; Gerger et al., 2007) to measure their rape myth beliefs, the *Homosexuality Attitude Scale* (HAS; Kite & Deaux, 1986) to measure whether levels of homophobia impacted judgments differently by complainant gender; and the *Just World Scale* (JWS; Dalbert et al., 1987) to measure the belief that in a just world people tend to get the outcome they deserve. This opinion is linked to increased victim blaming and assuming rape only happens to certain societal groups (e.g., Sleath & Woodhams, 2014; Strömwall et al., 2014; although see Russell & Hand, 2017).

The hypotheses were based on the assumption that the casting coach scenario would trigger rape myths. First, in comparison to when the complainant was not subsequently cast in the production, female complainant employment, but not male, was expected to reduce defendant guilt attributions, quantified by reduced guilty verdicts and recommended prison sentences. Second, a negative relationship between the delay in complainants reporting the crime to police and defendant guilt attributions was expected. Third, higher defendant guilt attributions were expected in the female than in the male complainant conditions, particularly from participants reporting higher HAS scores, consistent with higher levels of homophobia. Fourth, these effects were expected to be underpinned by a negative relationship between rape myth belief levels as measured by the AMMSA, and defendant guilt attributions. Fifth, a negative relationship was also expected between belief in a just world and defendant guilt attributions. As recruitment levels were high, participant gender was also included as a factor, and female participant-jurors were additionally expected to deliver harsher judgments than males, with the latter predicted to be more likely to endorse rape myths and to display higher levels of homophobia.

## Method

### Design

This study was approved by the School of Human Sciences Research Ethics Panel of the University of Greenwich. In a 2 (participant-juror gender: female, male) x 2 (complainant gender: female, male) x 2 (employment: job, no job) x 3 (reporting delay to police: 1 day, 6 months, 10 years) between-subjects design, participants were randomly assigned to view one of 12 videoed testimonies of actor-complainants describing a casting couch rape scenario. The dependent variables were verdict (guilty, not guilty) and sentence length (0–30 years). Scores on the AMMSA (Gerger et al., 2007), the JWS (Dalbert et al., 1987), and the HAS (Kite & Deaux, 1986) were also correlated with the dependent variables.

### Participants

Adult participants were recruited via social media and snowballing. Inclusion criteria closely matched UK jury eligibility in that participants were required to be aged 18–75 and resident in the UK for at least 5 years (Gov.UK, 2017). Note that adults may not be eligible for jury service if suffering mental health disorders or if they have committed a recent serious crime. Participants were not asked to exclude themselves on this basis. In total, 1,147 clicked on the link. However, many dropped out before completing all scales (*n* = 240), and only those who finished the research are included in analyses (*n* = 907, male = 322, female = 576, no response = 9; 18–25 years = 184 (20.3%), 25–35 = 269 (29.7%), 35–45 = 217 (23.9%), 45–55 = 141 (15.5%), 56+ = 91 (10.9%), no response = 5; white-Caucasian = 768 (84.7%), Asian/British-Asian = 38 (4.2%), Black-African/Afro-Caribbean = 15 (1.7%), and other ethnicity = 86). Except for an overrepresentation of females (expected ≈ 50%), the demographic profile roughly matches the UK population (Gov.UK, 2018).

### Materials and Procedure

After entering the survey on Qualtrics, being warned of the content’s sensitivity, and being asked to place themselves in the role of a juror, participants provided informed consent and demographic information before being randomly assigned to one of the 12 conditions and viewing the associated complainant’s video.

#### Complainant video testimony.

The video testimony was designed to replicate conditions of *Achieving Best Evidence* interviews (Judicial Studies Board, n.d.), the standard format for initial complainant testimony for courts in England and Wales (Ministry of Justice, 2011). The fictional script was based on amalgamated press reports of casting couch claims and incorporated the following factors. First, an actor “Sam” attends a meeting with the aim of being cast in a production. Second, the person they are meeting—a producer named “Jonathan”—has a notorious reputation as a serial sexual harasser which Sam obliquely refers to in their testimony. Third, the meeting takes place in daylight/office hours. Fourth, there is a description of Jonathan attacking Sam which meets the legal definition of rape.

Three additional components varied. For the *employment* factor, Sam states whether they got the acting job or not. For the *time taken to report* factor, Sam states it took 1 day, 6 months, or 10 years to report the rape to the police. Finally, for the *complainant gender* factor, white-Caucasian actors (one female: 39 years old, one male: 33 years old) were recruited, rehearsed, and filmed. The 12 videos (2 min 18s to 2 min 55s) were edited as best as possible to ensure equivalent intonation and emphasis, with the only points of divergence being the independent variables.^i^iThe full script (Annex A), written judicial guidance (Annex B) and with full informed consent the 12 actor videos (Annex C), together with the raw data (Annex D) are available in the supplementary link (https://doi.org/10.17605/OSF.IO/M8ZQ5).

Participants then read judicial advice adapted from the *Crown Court Bench Book* (Judicial Studies Board, n.d.) describing in layman’s terms the legal definition of consent, before answering the question, “Do you believe Jonathan is guilty of rape?” (yes, no), and providing a recommended sentence length on a sliding scale from 0 to 30 years after reading, “Jonathan was found guilty by the jury. Please indicate below the sentence length you would give Jonathan for this crime.” Thirty years as a maximum sentence is approximately double the expected starting point sentence for a serial rapist in England and Wales (Sentencing Council, 2013).

Participants then completed three scales.

##### Acceptance of Modern Myths About Sexual Aggression Scale (AMMSA) (English translation: Gerger et al., 2007).

This 30-item 7-point Likert scale (1 = strongly disagree to 7 = strongly agree) updates two previous rape myth acceptance scales (Burt, 1980; Payne et al., 1999) designed to address rape myth views becoming more nuanced, and thus requiring greater language subtlety (e.g., “To get custody of their children, women often falsely accuse their ex-husband of a tendency towards sexual violence”). High scores indicate highs level of rape myth acceptance. Scale reliability was high (Cronbach’s α = .93).

##### The Homosexuality Attitude Scale (Kite & Deaux, 1986).

On this 21-item 5-point Likert scale (1 = strongly disagree to 5 = strongly disagree^ii^iiAs recommended by the authors, item 2 from the original paper was excluded.; i.e., “The love between two males or two females is quite different from the love between two persons of the opposite sex”), low scores are normally indicative of high homophobic levels. Ten items are reverse scored. For clarity, high scores here were indicative of high levels of homophobia. Scale reliability was high (Cronbach’s α = .90).

##### The Just World Scale (Dalbert et al., 1987).

The JWS is derived from Just World Theory (Lerner, 1980), or the belief that the world is a just place, and people get outcomes they deserve. The original test comprised 14 items in two subscales (e.g., “I believe that, by and large, people get what they deserve”). However, the six-item 6-point Likert sub-scale (1 = strongly disagree to 6 = strongly disagree) used here measures “general belief in a just world” (see justification from Dalbert, 1999; Yu et al., 2018). Scores correlate with the rape myths of victim blaming and assuming rape only happens to certain groups. High scores indicate adherence to just world principles. Reliability was good (Cronbach’s α = .83).

Finally, participants rated the believability of the video testimony and scenario from 0 (completely unbelievable) to 100 (completely believable) and were debriefed. Note that due to experimenter error, some participants did not see this question, while others declined to respond (*n* = 119). Failure to provide a response did not differ by condition (*p* > .2).

## Results

Data were analyzed on SPSS using a significance level of α = .05. The Bonferroni correction was employed for post hoc analyses. Table 1 displays rates of guilty verdicts and mean recommended sentence lengths by experimental condition. After watching the video testimony, 73.6% of participants delivered a guilty verdict, recommending mean prison sentences of 12.26 years (*SD* = 7.39; range 0–30 years).

**Table 1.** Mock-Juror Verdicts and Mean Recommended Sentence Lengths as a Function of Complainant Gender, Employment, and Delay in Complainant’s Reporting Incident to Police.

				Guilty Verdicts	Sentence Lengths (Years)	
Complainant Gender	Employment	Reporting Delay	*n*	%	*M*	*SD*
Male	Job	1 day	72	76.4	11.58	6.44
		6 months	74	67.6	12.40	7.40
		10 years	77	80.5	13.26	7.95
	No job	1 day	74	71.6	11.97	7.86
		6 months	79	81.0	11.85	6.18
		10 years	78	69.2	13.13	8.19
Total			454	74.5	12.37	7.37
Female	Job	1 day	78	74.4	10.96	7.02
		6 months	82	68.3	12.20	8.14
		10 years	71	73.2	12.00	6.60
	No job	1 day	70	78.6	12.50	7.49
		6 months	76	64.5	12.21	7.66
		10 years	76	79.0	13.11	7.48
Total			453	72.9	12.15	7.42

*Note.* Participants not providing gender data are not included in this table (*n* = 11).

### Video Believability

With the exclusion of participants not providing gender information (*n* = 11) or a response (*n* = 117), a 2 (participant gender: female, male) x 2 (complainant gender: female, male) x 2 (employment: job, no job) x 3 (reporting delay: 1 day, 6 months, 10 years) between-subjects ANOVA on believability ratings revealed no effects or interactions (*p* ≥ .2), suggesting each condition’s scenario was equally convincing.

### Verdicts

To evaluate the impact of experimental conditions, a 2 (verdict: guilty, not guilty) x 2 (participant gender) x 2 (complainant gender) x 2 (employment) x 3 (reporting delay) backward elimination hierarchical log-linear analysis (probability for removal *p* < .05) revealed a significant four-way interaction between verdict, complainant gender, employability, and reporting delay, χ^2^(2) = 6.87, *p* = .032, and a significant two-way interaction between verdict and participant gender, χ^2^(1) = 29.81, *p* < .001; females (79.7%) delivered more guilty verdicts than males (62.7%).

Analyses on the three-way simple interaction effects as a function of employment found no effects when the complainant was cast in the performance (*p* > .2). However, when they did not gain the role, the interaction between verdict, complainant gender, and delay in reporting an offence was significant, χ^2^(1) = 8.25, *p* < .05. Bonferroni-corrected analyses on this interaction found no reliable post hoc significant effects (*p* > .05), and this possibly spurious non-significant interaction appeared driven by slightly higher guilty verdict rates in the male complainant (80.5%) compared to the female complainant (64.5%) condition after delays of six months only.

### Recommended Prison Sentence Lengths

A 2 (participant gender) x 2 (complainant gender) x 2 (perceived benefit) x 3 (reporting delay) between-subjects ANOVA on sentence lengths revealed only a significant main effect of participant gender, *F*(1, 874) = 4.48, *p* = .035, η^2^ = .005 (all other effects *p* > .195). Females (*M* = 12.64, *SD* = 7.47) provided longer sentence recommendations than males (*M* = 11.56, *SD* = 7.12).

In summary, the combined verdicts and sentences analyses supported the hypothesis that female mock-jurors would deliver harsher judgments than males. However, no reliable support was found for defendant guilt attributions being impacted by reporting delays, complainant gender, or whether the complainant was cast in the production or not. This is evaluated in the discussion.

### Gender Effects on Attributional Scales

Three independent-measures *t*-tests comparing male and female scores on the three attributional scales are reported in Table 2. These showed that as expected, males were significantly more accepting of rape myths and more homophobic. However, no JWS score differences were found.

**Table 2.** Mean Scale Scores by Gender on the AMMSA (Out of 7), HAS (Out of 5) and JWS (Out of 6), and Results of Independent-Measures *T*-Test Comparing Males and Females on These Scales.

*n*	Males		Females					
	322		576					
	*M*	*SD*	*M*	*SD*	*df*	*t*	*p*	*Cohen’s d*
AMMSA	3.13	0.91	2.70	0.89	896	–6.90	< .001	0.48
HAS	1.71	0.57	1.54	0.51	602.65	–4.56	< .001	0.31
JWS	3.09	1.08	3.09	0.92	581.58	<0.01	> .2	< 0.01

### Correlational Analyses

Table 3 depicts correlation coefficients between the primary variables. Verdicts and sentences correlated positively and moderately with scenario believability, and weakly but significantly with sentence length, AMMSA, and HAS scores. The strongest correlation however was between AMMSA and HAS scores, suggesting strong rape myth beliefs are associated with higher levels of homophobia. JWS scores also correlated weakly with AMMSA and HAS scores. However, in contrast to hypotheses, scores on the JWS bore no relationship with verdicts and sentences.

**Table 3.** Pearson’s Correlation Coefficients Between Primary Measures (*n* = 983).

	Sentence	Believability	AMMSA	HAS	JWS
Verdict	.20 *	.42 *	–.19 *	–.19 *	–.02
Sentence		.15 *	–.16 *	–.09 *	–.05
Believability			–.13	–.09 *	–.02
AMMSA				.47 *	.31 *
HAS					.17 *

*Note.* *p < .01.

### Predictors of Verdict

To examine the hypothesis that drivers of defendant guilt would differ by complainant gender, separate logistic regression analyses were performed on the by-complainant data. The verdict was the dependent variable, with predictors, scores on the AMMSA, HAS and JWS scales, participant gender (1 = female, 0 = male), and employment (1 = job, 0 = no job). To further explore the impact of the reporting delay effects found above, the dummy variable procedure was applied. The first compared effects of an immediate report with a delayed report (dummy variable 1: 1 = one-day, 0 = other delay); the second examined the effects associated with the six months delay condition (dummy variable 2: 1 = 6 months, 0 = other delay).

With the male complainant, 449 cases were analyzed and the significant full model, χ^2^(omnibus) *R*^2^ = 32.34, *df* = 7, *p* < .001, accounted for up to 10.3% (Nagelkerke *R*^2^) of the variance, with 74.6% of verdicts correctly classified. Only participant gender, B = –0.67, Wald (*df* = 1) = 8.33, *p* = .004, Exp B = 0.51 (95% CI = 0.33–0.81), and AMSSA scores, B = –0.36, Wald (*df* = 1) = 6.18, *p* = .013, Exp B = 1.43 (95% CI = 1.08–1.91), significantly and negatively. HAS scores, B = –0.43, Wald (*df* = 1) = 3.53, *p* = .060, Exp B = 1.53 (95% CI = 0.98–2.40) unexpectedly did not significantly predict verdicts (other predictors: *p* > .15).

With the female complainant, 449 cases were included, the full model was also significant, χ^2^(omnibus) *R*^2^ = 33.80, *df* = 7, *p* < .001, accounting for up to 10.5% (Nagelkerke *R*^2^) of the variance, with 72.6% of verdicts correctly classified. Only participant gender, B = –0.71, Wald (*df* = 1) = 9.74, *p* = .002, Exp B = 0.49 (95% CI = 0.31–0.77), and HAS scores, B = –0.49, Wald (*df* = 1) = 5.51, *p* = .019, Exp B = 1.64 (95% CI = 1.09–2.47) significantly predicted verdicts (other predictors: *p* > .69).

In summary, as predicted, regardless of complainant gender, females were more likely to deliver guilty verdicts. With the male complainant, higher rates of guilty verdicts were also associated with lower rape myth acceptance. When the complainant was female, higher homophobic attitudes were surprisingly associated with lower guilty verdict rates.

### Predictors of Sentence Length

Two multiple regressions with sentence as the dependent variable, were also conducted with complainant gender data separated. Predictors were AMMSA, HAS, and JWS scores, verdict (1 = guilty, 0 = not guilty), participant gender, complainant gender, employment, delay dummy variable 1 (1 = one-day, 0 = other delay), and delay dummy variable 2 (1 = 6 months, 0 = other delay).^iii^iiiAll assumptions were met (tolerance = 0.72–1.00, variance inflation factor (VIF) = 1.00–1.48).

Both models were significant, explaining, 6.7% = male, *R*^2^_Adjusted_ = .05, *F*(8, 440) = 3.96, *p* < .001; and 5.7% = female, *R*^2^_Adjusted_ = .04, *F*(8, 440) = 3.34, *p* = .001, of the variance respectively.

With the male complainant, only verdict, β = 4.51, *t* = 4.05, *p* < .001, 95% CI = 1.53–4.70, and AMSSA scores, β = 3.12, *t* = –2.67, *p* = .008, 95% CI = 0.31–2.04 significantly predicted sentence lengths.

With the female complainant, only verdict was significant, β = 2.84, *t* = 3.57, *p* < .001, 95% CI = 1.28–4.41). All other predictors were nonsignificant (*p* ≥ .088).

In summary, mean sentences were nearly three years longer after individual guilty (*M* = 13.34, *SD* = 7.31) than not guilty verdicts (*M* = 10.63, *SD* = 8.16), not surprisingly, as a participant providing an individual not guilty verdict would have shortly afterwards read that the jury as a whole had found the defendant guilty. They may still have disagreed with this proposition and recommended a less harsh sentence. Furthermore, rape myth beliefs were negatively predictive of sentences associated with the male complainant only.

## Discussion

This study examined factors commonly linked to quid pro quo harassment within the casting couch scenario, in which a notorious male entertainments industry figure allegedly rapes a complainant, whose original aim for meeting was to secure an acting role. Participant recruitment (*n* = 907) provided sufficient statistical power, and as expected, with strong effect sizes, female participant-jurors (79.7%) were significantly more likely than males (62.7%) to deliver a guilty verdict, and to recommend longer prison sentences (*M* = 12.6 *vs*. 11.6 years). Consistent with previous research (e.g., McGee et al., 2011), and as predicted, these effects were linked to higher AMSSA-measured (Gerger et al., 2007) rape myth beliefs, which were found to be significantly higher in males. However, no consistent effects of reporting delay, complainant gender, or whether the complainant gained employment or not were revealed here.

There was, however, a significant interaction between perceived benefit from gaining employment, length of delay before reporting the alleged offence to the police, and complainant gender on verdicts. Length of delay or complainant gender had no impact if the complainant was cast in the production. However, there was a significant interaction in the condition in which the complainant delayed reporting the incident for six months and they did not secure a role. Bonferroni-corrected post hoc analyses were not significant, and the interaction effect may have been spuriously driven by not guilty verdict rates being factually, but not significantly higher when the complainant was male (81.0%) than female (64.5%). These results directly contrast with assumptions of Sexual Economics Theory (Baumeister & Vohs, 2004) that female sexuality has more inherent value than males. This theory would predict that failure for the female complainant to be compensated by an offer of employment by the defendant, would, in comparison to a male complainant, have been expected to generate higher levels of guilt attributions towards that defendant. Participant scenario believability ratings did not differ by randomly allocated condition, ruling out acting variation as an explanation for these anomalous effects. Nevertheless, effect sizes driving these differences were small, and replication is required before any assumptions can be made as to why emerging effects were found in this specific condition.

Complainant gender-specific rape myth belief and homophobic attitude levels did predict verdicts and sentences. With the male complainant, rape myth scores on the AMMSA (Gerger et al., 2007) significantly and negatively predicted guilty verdict rates and sentences; not an unexpected result given the myth that “real rape” only impacts females. However, scores on the Homosexuality Attitudes Scale (HAS) scale did not predict verdicts associated with the male complainant.

In contrast, only participant gender and HAS scores (negatively) were significant predictors of guilty verdicts with the female complainant. No AMMSA (Gerger et al., 2007) effects were found, suggesting that rape myth beliefs did not have any additional impact on verdicts beyond the already strong participant gender effects. The impact of homophobic attitudes on participant-juror judgments in a case in which the alleged rape involved a heterosexual male-on-female attack was somewhat surprising, particularly as this factor was, unlike expectations, not significant for the male complainant (*p* = .060). Harsher defendant judgments had been expected in the female than in the male complainant conditions (van der Bruggen & Grubb, 2014), particularly by participants displaying high levels of homophobia. As rape myth beliefs and homophobic attitudes were positively and moderately correlated, with males scoring significantly higher on both, it suggests that these attitudes may reflect a general negative bias towards females making complaints of rape in the casting coach scenario. Indeed, other factors such as right-wing authoritarianism and general prejudicial attitudes (e.g., Whitley, 1999), and beliefs in traditional gender roles (Kerns & Fine, 1994) tend to positively correlate with homophobic attitudes. As with other features of the current research, this lack of consistency with previous rape myth research suggests that different stereotypical influences may operate on attributions towards quid pro quo harassment associated with the casting couch scenario.

One possible explanation is that the mean recommended prison sentence across all conditions of 12.3 years was far higher than statutory rape sentencing guidelines for a single offence of five years in England and Wales (Crown Prosecution Service, 2012). In the videoed testimony, the defendant is described as having a “reputation.” As such, even though asked to judge a single case, participants may have assumed multiple offences had been committed. For this, 15 years imprisonment is common in the UK (Sentencing Council, 2013). This ambiguity may also explain the exceptionally wide variety of sentence recommendations made by participants regardless of verdict, as well as the (relatively) weak correlation between the verdicts and sentences (*r* = .20). Indeed, recommended sentences varied from 0–30 years after both guilty and not guilty verdicts representing very wide differences in opinions as to a suitable punishment.

Unlike previous research (Dinos et al., 2015; Sleath & Woodhams, 2014), except for weak correlations with AMMSA (Gerger et al., 2007) and HAS (Kite & Deaux, 1986) scores, there were no effects associated with JWS (Dalbert et al., 1987) scores on any outcome. Research on other crimes tends to find a strong relationship between offender blaming and strong belief in a just world. The distinctiveness of rape is that complainant behavior, rather than defendant behavior is often the principal focus, both in media reports and in trials (Dinos et al., 2015). If the “reputation” of the defendant was interpreted by participant-jurors as a pattern of dangerous behavior, and the complainant willingly put themselves into a risky situation, this may have been the most salient driver of guilt attributions here—and this did not differ by condition. This idea of a complainant choosing to put themselves in a position of vulnerability (by getting drunk) was explored by Gray (2015). Many participants in that research attributed more blame to the complainant for her rape. By extension, an actor willingly meeting with a high-status high-risk industry figure may be seen in the same light. Nevertheless, scale completion in the current study may have been biased by having already delivered a verdict. To rule out such potential order effects, variable order could be counterbalanced in future research, with some participants completing scales prior to reading the case materials and delivering verdicts.

There were also some limitations to this research in terms of diversity. The current research focused on White complainants only. One feature of the Casting Couch scenario, and quid pro quo sexual harassment in general, particularly in the USA, is that a high proportion of survivors are from non-White backgrounds. Indeed, Black women are three times more likely than White women to be victims of workplace sexual harassment in the USA (Rossie et al., 2018). The ethnicity of complainants of police reported rapes in the UK closely matches that of the general population (Waterhouse et al., 2016). However, worryingly, sexual violence services in the UK show that women identifying as Black or other minority ethnicities are at a higher risk of sexual violence, suggesting many do not report crimes to the police (e.g., Rape Crisis, 2019). Despite the inclusion of a male rape victim condition—also under-researched—the focus on white complainants in the current research therefore represents a clear limitation in terms of diversity, and furthers the marginalization experienced by a large number of complainants (Onwuachi-Willig, 2018).

The current research also has diversity implications in terms of participation. The juror-participant sample, although UK jury-eligible, was proportionally dominated by females (64%), a probable consequence of higher interest in this topic. A roughly equal split would be expected of a real jury, as virtually any UK adult may be randomly selected from to sit on a jury (Gov.UK, 2017). On the other hand, most juror decision-making research recruits students, and only 20% of the participants in the current research were of the typical 18–25-year-old student age group. Furthermore, the ethnic constitution of the sample closely matched that of the UK population, in which approximately 86% of the population is White (Gov.UK, 2018). As such, the slight gender imbalance may have been outweighed by participant’s representative diverse age and ethnicity profile. Related to this is that although jury procedures may differ in the UK from those in other countries, the decision-making of individual jurors with pre-existing attitudes in such cases is unlikely to substantially differ internationally. Therefore, these results are likely applicable outside the UK.

The video testimony was written to meet the legal definition of rape in the UK, while judicial advice was provided to participants describing the law in relation to consent. However, no mitigating defendant statement was included, to ensure participants’ focus was directed at variables of interest. As approximately one-in-four participants delivered a not guilty verdict, it is clear that the scenario provided sufficient ambiguity for assessment of included variables, although there is always a risk that significant effects are obscured with such overall consistently high rates of guilty verdicts. Nevertheless, a real jury would deliberate as a group before delivering a verdict. This also limits study validity, although strong pre-deliberation majorities tend to prevail, and only 13% of jurors change their initial verdict choice following deliberation (Booth et al., 2017).

Real juries would also likely be exposed to rape myths being “weaponized” on behalf of the defendant (Dinos et al., 2015). The Crown Prosecution Service (2012) and the Judicial Studies Board (n.d.) have produced guidance (for prosecutors and judges, respectively) on how to counter effects. Although this guidance may not be regularly employed by judges or prosecutors (Temkin et al., 2018), it is designed to encourage the jury to disregard beliefs and to challenge defense counsel if rape myths are used. From the results here, attributions towards the Casting Couch Scenario are clearly impacted by rape myths, and future research should also evaluate myth-reducing intervention impact within this context.

## Conclusions

This study on the Casting Couch scenario revealed that male participant-jurors were significantly more likely to deliver a not guilty verdict than females, a result linked to higher levels of rape myth acceptance. However, evaluation of factors reported in the media to be common components of the casting coach scenario, including whether the complainant gained the acting role or not, and delay in reporting the incident to the police did not reveal effects consistent with expectations. Further research should be directed at this topic as it may have implications outside the entertainment industry, particularly in other occupational settings in which senior management may similarly sexually exploit their position of power over more junior employees from different ethnic backgrounds (Rossie et al., 2018). The attitudes and biases identified in participants in the current study are likely shared with many individuals working within the criminal justice system internationally. It is important that policymakers worldwide are aware when there may be miscarriage of justice risks.

## Author Biographies

**Shona Mcintosh**, MSc, was an MSc Psychology (Conversion) student at the University of Greenwich. This project was part of her distinction level MSc dissertation while a student in the School of Human Sciences at the University of Greenwich.

**Josh P Davis,** BSc, MSc, PhD, is a Reader in Applied Psychology at the University of Greenwich in London. ​His teaching and research are strongly focused on police investigative procedures, eyewitness identification, and the reduction of miscarriages of justice. His first co-edited book with Tim Valentine *Forensic Facial Identification: Theory and Practice of Identification from Eyewitnesses, Composites and CCTV* (Wiley Blackwell) was published in 2015.
